# Supplementary material for: From sequence to enzyme mechanism using multi-label machine learning
Source: BMC Bioinformatics. 2014 May 19;15:150. doi: 10.1186/1471-2105-15-150 (PMC4229970; doi:10.1186/1471-2105-15-150)
Supplement: Additional file 2 — Java code of ml2db. Additional file ml2db_code.tar.gz contains the Java source code to run the multi-label machine learning experiments and save the results to database. The code’s Javadoc is included. [file 1471-2105-15-150-S2.zip › additional file 2/ml2db/ml2db/doc/main/ml2db/mulan/learn/traintest/class-use/TrainTestExperimenter.html]

Uses of Class main.ml2db.mulan.learn.traintest.TrainTestExperimenter


JavaScript is disabled on your browser.


- Overview
- Package
- Class
- Use
- Tree
- Deprecated
- Index
- Help

- Prev
- Next

- Frames
- No Frames

- All Classes

## Uses of Class main.ml2db.mulan.learn.traintest.TrainTestExperimenter

- Packages that use TrainTestExperimenter

  | Package | Description |
  | --- | --- |
  |  |  |
  | --- | --- |
  | main.ml2db.al.learning |  |
  | main.ml2db.mulan.learn.traintest |  |
  | main.ml2db.mulan.predict |  |
  | test.ml2db.mulan.learn.traintest |  |
- - ### Uses of TrainTestExperimenter in main.ml2db.al.learning

    Subclasses of TrainTestExperimenter in main.ml2db.al.learning

    | Modifier and Type | Class and Description |
    | --- | --- |
    |  |  |
    | --- | --- |
    | `class` | `AlExperimenter` Class |
  - ### Uses of TrainTestExperimenter in main.ml2db.mulan.learn.traintest

    Subclasses of TrainTestExperimenter in main.ml2db.mulan.learn.traintest

    | Modifier and Type | Class and Description |
    | --- | --- |
    |  |  |
    | --- | --- |
    | `class` | `TrainTestExperimenterSerialized` Executes a train/test evaluation using a previously trained model saved on file. |

    Methods in main.ml2db.mulan.learn.traintest that return TrainTestExperimenter

    | Modifier and Type | Method and Description |
    | --- | --- |
    |  |  |
    | --- | --- |
    | `TrainTestExperimenter` | TrainTestEvaluator.`getExperimenter()` |
    | `TrainTestExperimenter` | TrainTestOutputManager.`getExperimenter()` |

    Methods in main.ml2db.mulan.learn.traintest with parameters of type TrainTestExperimenter

    | Modifier and Type | Method and Description |
    | --- | --- |
    |  |  |
    | --- | --- |
    | `void` | TrainTestExperimenter.`setInputManager(int trainArffRecordId, DbManagers dbMan, TrainTestExperimenter trainTestExperimenter)` |

    Constructors in main.ml2db.mulan.learn.traintest with parameters of type TrainTestExperimenter

    Constructor and Description

    `TrainTestEvaluator(TrainTestExperimenter experimenter)`

    `TrainTestEvaluatorSerialised(TrainTestExperimenter mulanTrainTestExperimenter,
    java.lang.String serialisedTrainedModel)`

    `TrainTestInputManager(IdMultiLabelInstances trainSet,
    IdMultiLabelInstances testSet,
    TrainTestExperimenter experimenter)`

    Gets the instances as object.

    `TrainTestInputManager(IdMultiLabelInstances trainSet,
    TrainTestExperimenter experimenter)`

    Gets the instances as object.

    `TrainTestInputManager(int trainArffRecordId,
    DbManagers dbMan,
    TrainTestExperimenter trainTestExperimenter)`

    `TrainTestInputManager(int trainArffRecordId,
    int testArffRecordId,
    DbManagers dbMan,
    TrainTestExperimenter experimenter)`

    `TrainTestOutputManager(uk.ac.standrews.utils.main.database.DbManager resultsDbManager,
    java.lang.String resultsFolder,
    uk.ac.standrews.utils.main.database.Table table,
    TrainTestExperimenter experiment)`
  - ### Uses of TrainTestExperimenter in main.ml2db.mulan.predict

    Constructors in main.ml2db.mulan.predict with parameters of type TrainTestExperimenter

    Constructor and Description

    `PredictionsSaver(TrainTestExperimenter ttExpManager,
    mulan.data.MultiLabelInstances trainSet,
    mulan.data.MultiLabelInstances testSet,
    mulan.classifier.MultiLabelLearner trainedLearner,
    boolean saveTrueNegatives)`

    Predictor hooked into a train-test evaluation experiment.
  - ### Uses of TrainTestExperimenter in test.ml2db.mulan.learn.traintest

    Methods in test.ml2db.mulan.learn.traintest that return TrainTestExperimenter

    | Modifier and Type | Method and Description |
    | --- | --- |
    |  |  |
    | --- | --- |
    | `static TrainTestExperimenter` | TrainTestExperimenterTest.`getMulanTrainTestExperBRk1NN()` |
    | `static TrainTestExperimenter` | TrainTestExperimenterTest.`getMulanTrainTestExperBRk1NNById(int trainId, int testId)` |
    | `static TrainTestExperimenter` | TrainTestExperimenterTest.`getMulanTrainTestExperById(int trainId, int testId, MulanLearner learner)` |

- Overview
- Package
- Class
- Use
- Tree
- Deprecated
- Index
- Help

- Prev
- Next

- Frames
- No Frames

- All Classes
